# Oral Tofacitinib Therapy for the Effective Management of Netherton Syndrome

**DOI:** 10.7759/cureus.39349

**Published:** 2023-05-22

**Authors:** Ajay Dodeja, Kirtee Meshram, Sushil Pande, Manjiri Oke, Milind Borkar

**Affiliations:** 1 Dermatology, NKP Salve Institute of Medical Sciences and Research Centre and Lata Mangeshkar Hospital, Nagpur, IND

**Keywords:** atopic dermatitis, icthyosis linearis circumflexa, bamboo shaft hair, netherton syndrome, tofacitinib

## Abstract

An uncommon form of ichthyosiform erythroderma, Netherton Syndrome (NS) is inherited by an autosomal recessive pattern. Owing to eczematous skin lesions and the clinical features of atopy, NS is often initially diagnosed as atopic dermatitis. There are very few reports on NS in India. Hardly any case report or series that presents the use of biologicals for the treatment of NS reports the use of tofacitinib therapy. Therefore, it is essential to document such cases to promote further research to understand the underlying pathophysiology and find more effective treatments for the disease.

A three-year-old boy, the second issue of a non-consanguineous marriage reported a history of waxing and waning of generalized reddish-brown scaly plaques all over the body and recurrent infections since birth. Multiple annular erythematous, partially blanchable papules to plaques with double-edge scaling were observed most prominently on the trunk. There was a diagnostic dilemma among erythrokeratoderma variabilis (EKV), atopic dermatitis (AD), and ichthyosis linearis circumflexa (ILC).

The patient was administered betamethasone orally. However, there was no satisfactory relief or remission; therefore, oral tofacitinib therapy was initiated. The patient showed a good therapeutic response to oral tofacitinib at the dose of 0.3 mg/kg/day at the eighth-week follow-up.

## Introduction

Comel (1949) and Netherton (1950) first described the rare autosomal recessive condition known as Netherton syndrome (NS) (1958). As per Saleem et al., it consists of a triad, congenital ichthyosiform erythroderma (CIE) or ichthyosis linearis circumflexa (ILC), anomalies of the hair shaft, and atopic dermatitis (AD) [1]. Although ichthyosiform erythroderma typically manifests as ILC, it occasionally occurs in the CIE form in patients with NS. Trichorrhexis invaginata, pili torti, and trichorrhexis nodosa are hair shaft abnormalities seen [2]. With very few case reports on Netherton Syndrome from India, we report this as a case of NS initially mistreated as AD.

## Case presentation

**Figure 1:**
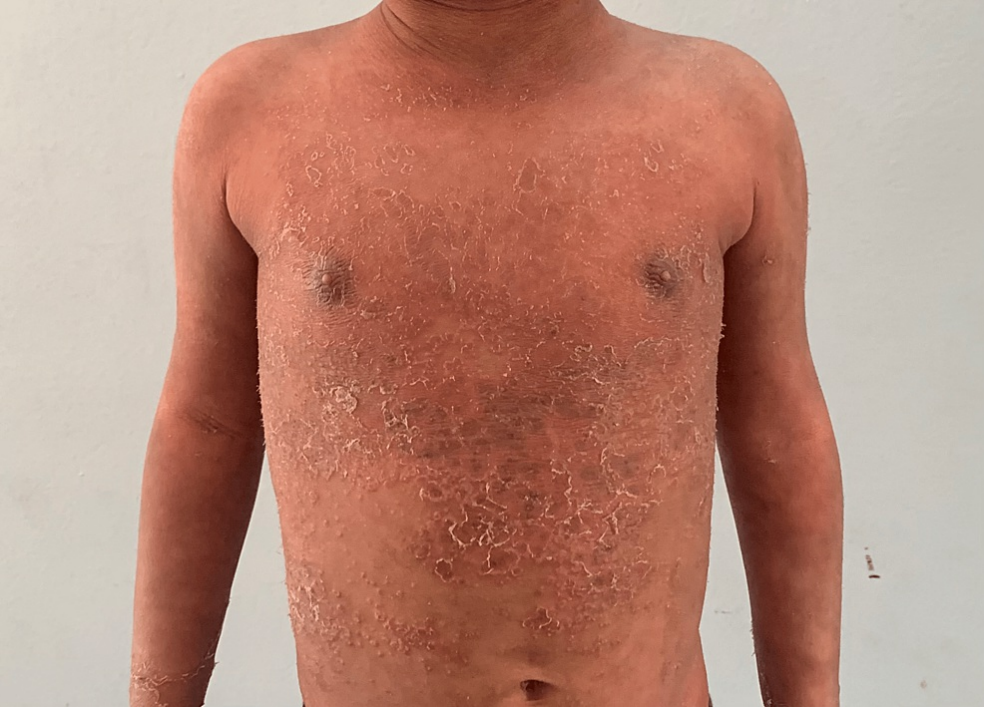
Generalized reddish-brown scaly plaques over trunk and upper limbs.

A three-year-old boy, the second child of a non-consanguineous marriage reported a history of waxing and waning of generalized reddish-brown scaly plaques all over the body and recurrent infections since birth (Figure 1).

**Figure 2:**
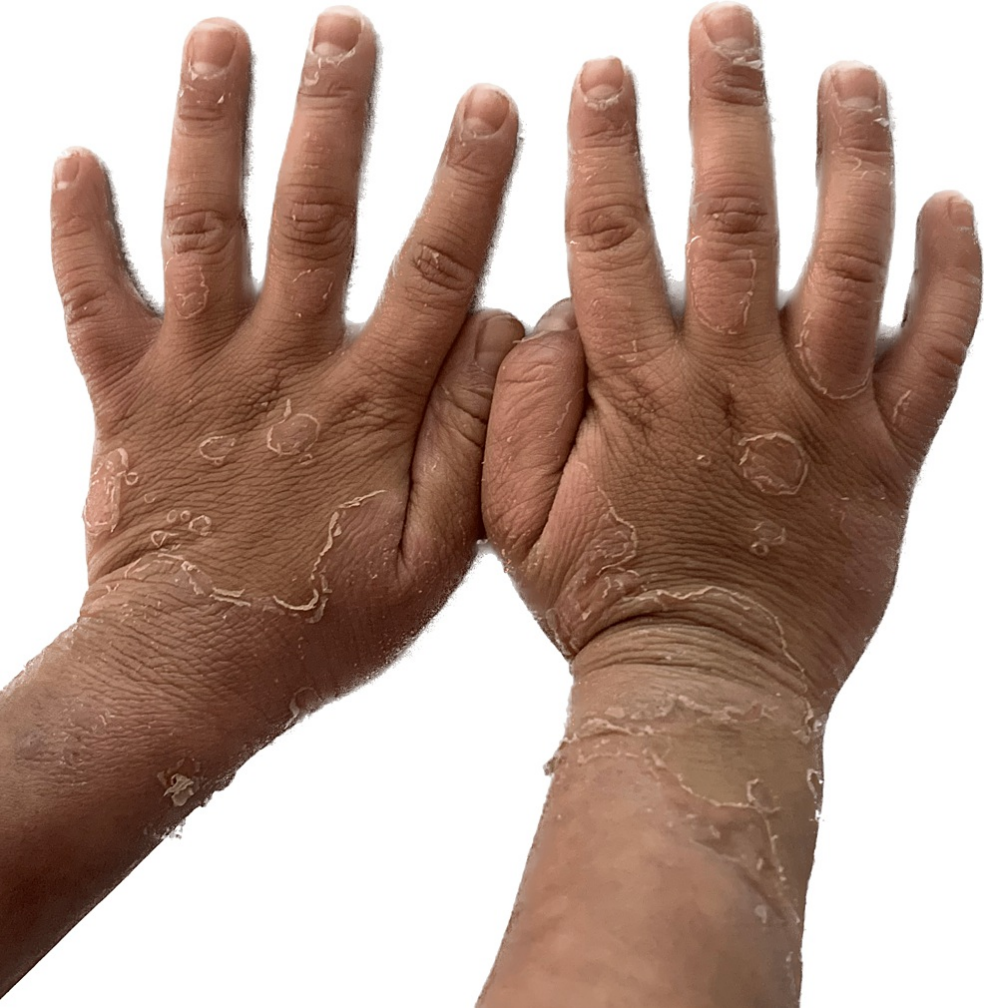
Double edge scaling.

Multiple annular erythematous partially blanchable papules to plaques with double-edge scaling were seen (Figure 2). Typical ILC-like lesions started appearing at 18 months. The child's parents did not give a history of breakage of hair. However, the hair growth rate was reported to be slower.

There was a diagnostic dilemma among erythrokeratoderma variabilis (EKV), AD, and ILC.

**Figure 3:**
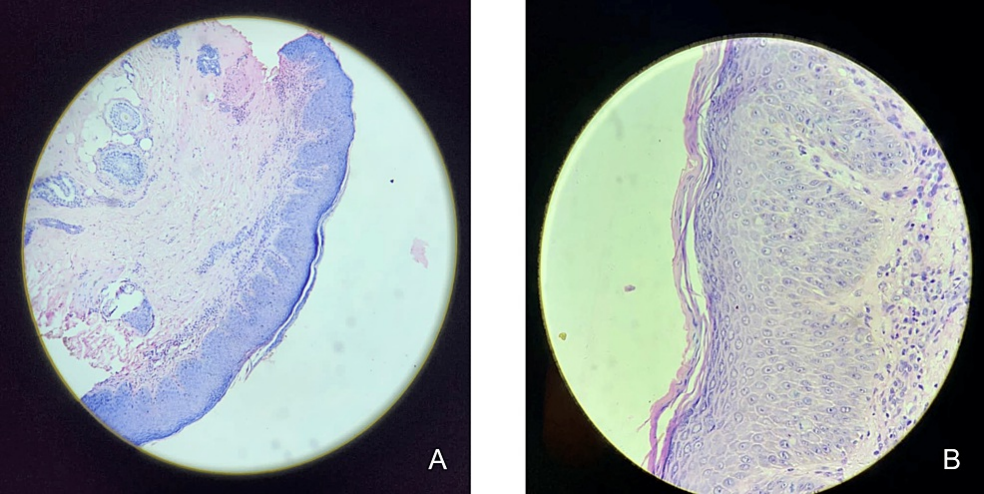
Histopathological examination showing hyperkeratosis with mild parakeratosis, spongiosis, acanthosis with focal bulbous rete ridges, and prominent granular cell layer at 10x (A) and 40x (B).

Histopathological examination of the skin biopsy showed hyperkeratosis with mild parakeratosis, spongiosis, acanthosis with focal bulbous rete ridges, and a prominent granular cell layer, suggesting AD (Figure 3).

**Figure 4:**
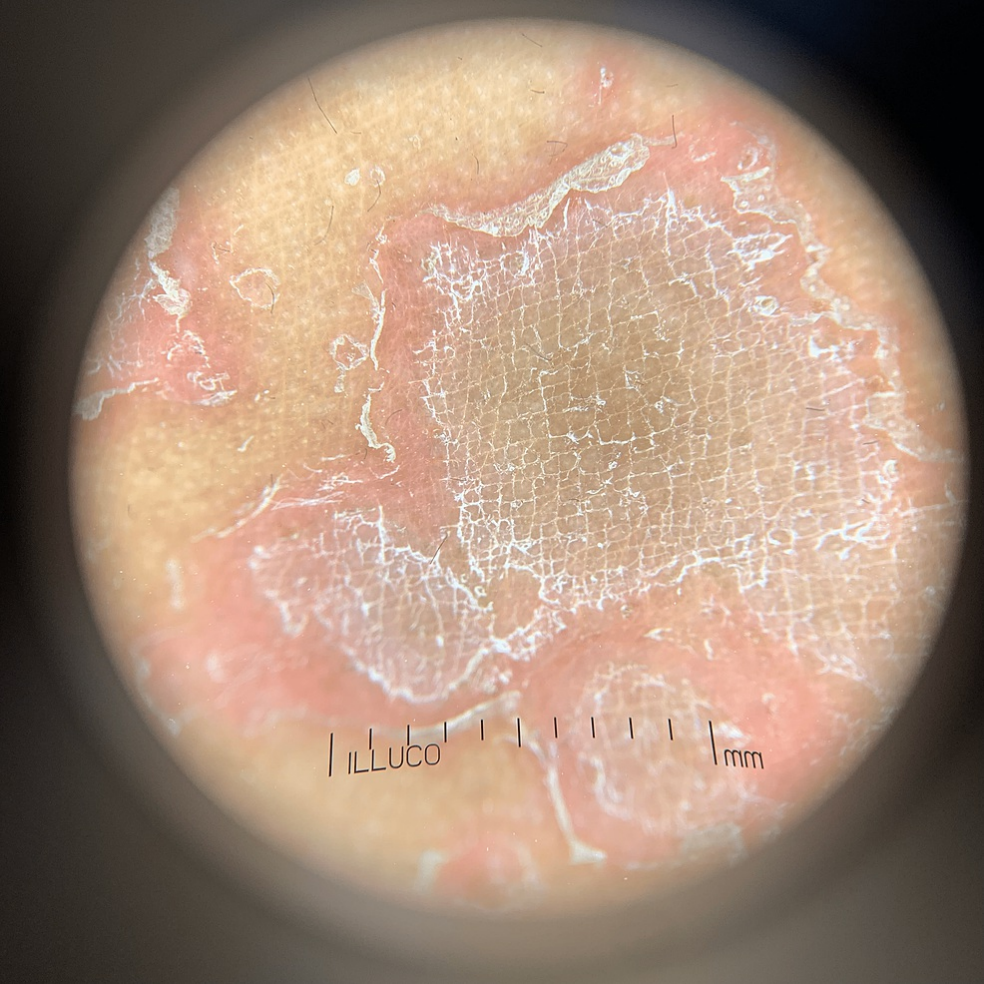
Erythematous plaque with central fish net-like scaling and serpiginous borders and double edge scaling seen on dermoscopy at 10x using polarized view.

Serum IgE levels were within normal limits. On dermoscopy, erythematous plaque with a central fish net-like scaling and serpiginous borders with double-edge scaling are seen (Figure 4).

**Figure 5:**
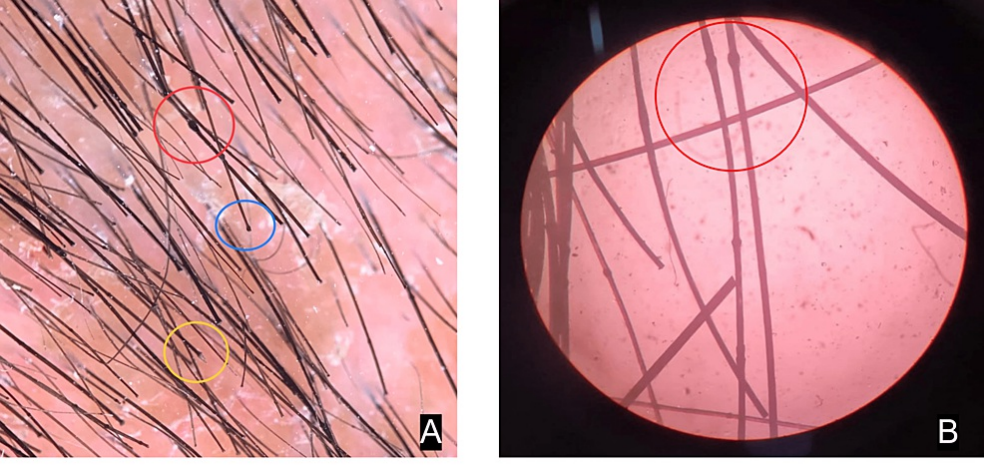
Bamboo shaft hair / trichorhexis invaginata demonstrated (red) on dermoscopy (A) and hair shaft microscopy (B). Flame sign (yellow) and golf tee sign (blue) seen on dermoscopy (A).

Bamboo shaft hair also known as trichorhexis invaginata (TI) was demonstrated on hair shaft microscopy as well as trichoscopy (Figure 5).

**Figure 6:**
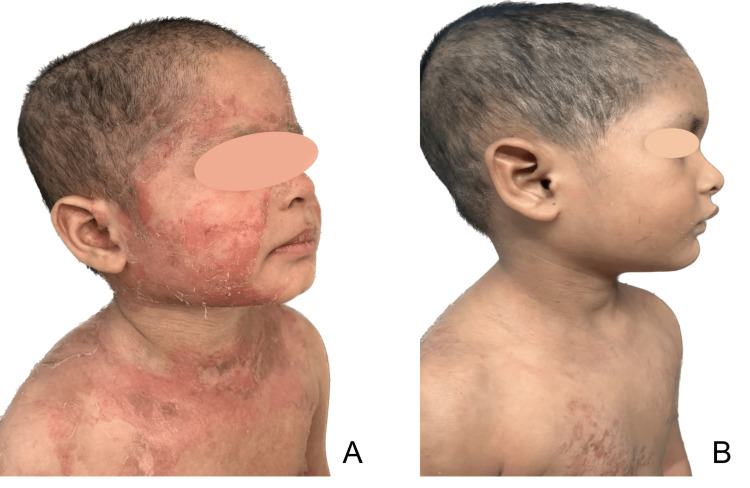
Cutaneous manifestation before (A) and 8 weeks after (B) starting oral tofacitinib therapy.

The patient was given oral betamethasone initially. However, there was no satisfactory relief or remission; therefore, oral tofacitinib therapy was planned. Interferon-gamma release assay (IGRA) was performed to rule out tuberculosis before initiation of tofacitinib therapy and was found to be negative. Currently, he is being treated with the help of the tablet tofacitinib 0.3mg/kg/day along with supportive treatment for eight weeks (Figure 6).

## Discussion

NS is an uncommon kind of erythroderma, that is inherited by an autosomal recessive gene. The precise incidence of NS is unknown; however, it is estimated to be 1/200,000 [3]. Epidermal proteases become hyperactive as a result of alterations in the serine protease inhibitor (SPINK5) gene, which is situated on chromosome 5q31-32 and promotes desquamation [1].

Through proteinase-activated receptor 2 (PAR2) signaling in keratinocytes, the unhindered action of epidermal proteases in the LEKTI-deficient epidermis of NS patients leads to the secretion of proinflammatory interleukin (IL)-8, tumor necrosis factor- (TNF-) and proallergic cytokines, thymic stromal lymphopoietin (TSLP) [4].

Erythroderma is typically present from birth, but due to a delay in hair growth, the diagnostic hair signs may not show up until later [5]. Clinically the hair is short, lusterless, brittle, and coarse. On microscopy and dermoscopy, it is noted that the proximal extremity of the hair shaft is invaginated by its distal segment. The hair of patients with NS breaks easily due to reduced disulfide bonds [6]. According to a term proposed by de Becker et al. [7] in 1995, the golf tee appearance results from hair that splits at the site of invagination. Goujon et al. [8] referred to a shattered hair shaft that has a protruding end as a "matchstick". DNA sequencing, demonstrating a germline SPINK5 mutation, supports the diagnosis. Nonetheless, its use in diagnosis is restricted due to the expense of DNA analysis [6].

Tofacitinib is a selective Janus kinase (JAK) inhibitor of the second generation that targets the JAK1 enzyme. It has FDA clearance for the management of juvenile polyarticular idiopathic arthritis (pcJIA) among kids older than two years old [9].

In a comprehensive study, Nouwen et al. [10] reviewed the various treatments implemented in 36 case series and reports of NS. Retinoids, prednisolone, cyclosporine, immunoglobulins, and biologicals were used as treatments. Where biologicals and immunoglobulins demonstrated the most encouraging outcomes, oral tofacitinib usage was not mentioned in any of the cases reported.

## Conclusions

NS has no effective etiological treatment available yet. Patients live with atopic diathesis along with cutaneous symptoms in varying severity throughout their life. TI, which is a pathognomic feature, is usually suggestive of the diagnosis. Therefore, a hair examination should be done early on to ensure a prompt and correct diagnosis. Due to the dearth of information on treatment methods in the literature, the most suitable customized treatment plan must be chalked out as per the clinical features of NS patients. We tried to keep the symptoms in check with the help of tofacitinib because its safety profile is better than that of systemic corticosteroids and other options.
